# Changes in Soil Microorganisms After Planting *Idesia polycarpa* in the Luohe River Basin

**DOI:** 10.3390/microorganisms14030646

**Published:** 2026-03-13

**Authors:** Xiaolong Hao, Qifei Cai, Tong Niu, Yingjian Niu, Zhongyu Wang, Zhen Liu, Yanmei Wang, Xiaodong Geng, Juan Wang, Yongyu Ren, Fangming Liu, Yaohui Liu, Li Dai, Zhi Li

**Affiliations:** 1 College of Forestry, Henan Agricultural University, Zhengzhou 450046, China; haoxiaohong@stu.henau.edu.cn (X.H.); cai_qifei@henau.edu.cn (Q.C.); niutong@stu.henau.edu.cn (T.N.); 19139976188@163.com (Y.N.); wzy159378@163.com (Z.W.); liuzhen@henau.edu.cn (Z.L.); wym@henau.edu.cn (Y.W.); xiaodonggeng@henau.edu.cn (X.G.); wangjuan0807@henau.edu.cn (J.W.); yongyuren_1128@henau.edu.cn (Y.R.); fangmingliu-0930@henau.edu.cn (F.L.); yaohuiliu@henau.edu.cn (Y.L.); 2 National Forestry and Grassland Administration Key Laboratory for Central Plains Forest Resources Cultivation, Henan Agricultural University, Zhengzhou 450046, China; 3 Henan Province Engineering Technology Research Center for Idesia, Zhengzhou 450046, China

**Keywords:** *Idesia polycarpa*, soil bacteria, soil physicochemical properties, soil microbial community diversity, Luohe River Basin

## Abstract

*Idesia polycarpa* ‘Yitong 2’ is a high-oil cultivar widely promoted in central China, yet field evidence on how soil bacterial communities respond during early plantation establishment remains limited. Here, we conducted fixed-site monitoring in a newly established ‘Yitong 2’ plantation in the Luohe River Basin (Henan, China). Bulk soil (0–30 cm) was collected before planting (March 2024) and at 3, 6 and 12 months after planting (June 2024, September 2024 and March 2025). Soil physicochemical properties were measured and bacterial communities were profiled by 16S rRNA gene (V3–V4) amplicon sequencing; functional potential was inferred using PICRUSt2. Available potassium increased significantly, whereas soil organic matter showed a decrease–recovery trajectory. Bacterial richness (Chao1) decreased after planting, while evenness increased; Shannon diversity remained stable. Community composition shifted directionally, with higher relative abundance of Pseudomonadota (formerly Proteobacteria) and reduced Acidobacteriota at later stages. PERMANOVA based on Bray–Curtis distances indicated significant temporal differences in community structure. RDA indicated that soil organic matter and bulk density were the primary drivers of community structural variation. Functionally, the overall metabolic framework remained stable, whereas pathways related to genetic information processing and metabolism exhibited significant differences (*p* < 0.05). By examining both intra-annual dynamics and inter-annual changes in soil bacteria and physicochemical properties following the planting of ‘Yitong 2’, this study clarifies patterns of soil property variation and trajectories of microbial community structure and functional potential, thereby providing a scientific basis for the establishment of high-quality *I. polycarpa* plantations and the sustainable development of soil ecosystems.

## 1. Introduction

*Idesia polycarpa*, a deciduous tree belonging to the genus *Idesia* of the family Flacourtiaceae, is characterized by strong adaptability, rapid growth, and high oil contents in its fruits [1]. It not only has timber and ornamental value, but also serves as an excellent emerging woody oil tree species [2]. The oil extracted from *Idesia polycarpa* is rich in various fatty acids, among which, linoleic acid is the most abundant component, accounting for more than 80% of the total fatty acids [3]. With favorable antioxidant capacity [4] and notable medicinal value [5,6], this tree species boasts broad prospects for development and utilization.

Soil serves as the material basis for plant growth, and its physicochemical properties directly affect vegetation development and the functions of soil ecosystems. As an important component of soil ecosystems, soil microorganisms drive nutrient cycling, organic matter transformation, and soil structure formation [7]. Meanwhile, soil microorganisms are extremely sensitive to environmental changes; soil type [8], vegetation type [9], land use pattern [10], and other factors exert significant impacts on their community structure and diversity. Plants regulate rhizosphere microbial communities through the input of root exudates and litter, whereas microorganisms feedback to plant growth via mechanisms such as promoting nutrient activation and enhancing plant stress resistance, thus forming a close feedback relationship between the two parties [11,12]. Therefore, the response of soil microbial communities is an important indicator for evaluating vegetation restoration and the sustainability of plantation ecosystems [13]. Soil bacteria are characterized by high diversity, large population size, and extremely wide distribution [14], and they can adapt to different environmental conditions by regulating their community structure [15,16]. They play a crucial role in soil carbon and nitrogen cycling and maintaining ecosystem stability, and profoundly affect the formation of soil fertility, decomposition of organic matter, regulation of nutrient cycling, suppression of pest and disease transmission, and regulation of plant growth and development [17,18]. Root exudates are a key source of carbon substrates and signaling compounds that plants use to regulate rhizosphere microbiome assembly, jointly shaping microbial community structure and activity through nutrient supply and chemical cues [19,20]. Their constituents typically include low-molecular-weight, readily degradable compounds such as soluble sugars, amino acids, and organic acids, as well as secondary metabolites such as phenolic acids, flavonoids, and volatiles/terpenoids; they also comprise high-molecular-weight components including mucilage, glycoproteins, and polysaccharides [21]. The composition and release intensity of root exudates often shift in a stage-specific manner with plant phenological progression and are strongly regulated by environmental factors; plants may increase the production of specific defense- and/or signaling-related exudates, thereby exerting stronger selective pressure on particular functional groups [19,22]. These temporal dynamics in root exudation not only regulate microbial diversity and community structure but may also further influence microbially mediated ecological functions, including carbon, nitrogen, and phosphorus cycling and the transformation of soil organic matter [23].

At present, studies on *Idesia polycarpa* have mostly focused on physiological and biochemical analysis [24,25], stress responses [26,27], and fruit quality analysis [28,29], whereas research on soil microbial communities is often confined to the description of single community characteristics [30,31]. Although some studies have examined the dynamic changes in *Idesia polycarpa* roots and soil nutrients [32] or differences in rhizosphere microbiota among stands of different ages [33], continuous observations and mechanistic investigations of the systematic feedback between *I. polycarpa* growth and soil microbial communities under field-scale plantation conditions remain scarce. Existing work has shown that rhizosphere bacterial community structures differ significantly between male and female plants during the flowering and fruit-maturation stages; the dominant phyla shift with phenological development from Actinobacteriota to Pseudomonadota, and bacterial diversity is significantly associated with soil pH, available potassium, and other factors. Functional prediction further indicates that metabolic pathways constitute the major functional category of rhizosphere microbiota. In addition, other studies have reported that different land-use types are characterized by distinct soil environmental conditions and corresponding differences in soil microbial community structure. Collectively, these findings suggest that shifts in microbial community composition may be closely linked to plant metabolic demands at different developmental stages and to soil nutrient status [30,31]. Accordingly, this study addresses the following questions: After *I. polycarpa* planting, do soil physicochemical properties and bacterial communities exhibit temporal variation and coordinated responses? Which soil environmental factors are the key drivers shaping bacterial community succession? How do bacterial metabolic functions respond to planting activities, and what mechanisms underpin their functional stability?

The Luohe River Basin lies within a transitional zone of the warm-temperate monsoon climate, characterized by complex landforms and confronted with pressing needs for ecological restoration and the development of the woody oil industry. Given the current paucity of continuous observations during the establishment stage of newly planted *Idesia polycarpa* under field conditions, this study focused on newly established plantations in this region. Using a one-year fixed-site field monitoring approach, we systematically analyzed intra-annual dynamics in soil physicochemical properties and soil bacterial community structure following *I. polycarpa* planting, compared soil ecological responses between pre-planting conditions and one year after planting, and explored the feedback relationships among soil factors, plant growth, and microbial communities. The findings provide a scientific basis for soil ecological management and sustainable operation of *I. polycarpa* plantations in the Luohe River Basin.

## 2. Materials and Methods

### 2.1. Overview of the Study Area

**Figure 1:**
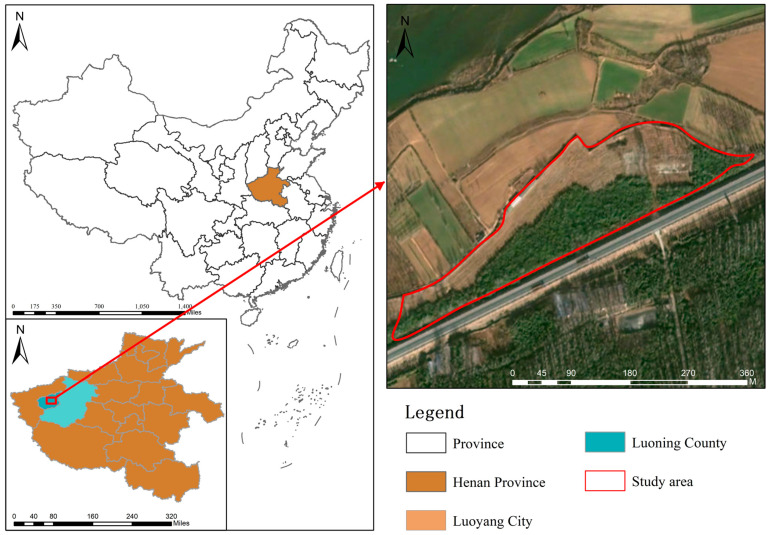
Location of the study site in the Luohe River Basin, Henan Province, China.

The study site is located in the section of the Luohe River Basin within Luoning County, Henan Province (between 34°05′ N and 34°38′ N, 111°08′ E and 111°49′ E) (Figure 1). Luoning County has a warm temperate continental monsoon climate with four distinct seasons, an annual frost-free period of 216 days, an annual average temperature of 13.7 °C, and an annual precipitation ranging from 600 to 800 mm. The Luohe River runs through the entire county from west to east, and the landform is characterized by the pattern of “seven parts mountains, two parts loess tablelands, and one part valleys”. This unique geomorphological pattern has fostered diverse ecosystem types and rich biodiversity [34]. Three-year-old *Idesia polycarpa* seedlings were planted in March 2024 with a planting spacing of 3 m × 4 m. Consistent tending and management measures were implemented in the forestland during the experimental period.

### 2.2. Soil Sample Collection and Preservation

To characterize the temporal dynamics of soil bacterial communities during the early establishment stage of *Idesia polycarpa* plantations, we conducted fixed-site monitoring at four time points: mid-March 2024 (0 months; pre-planting baseline), mid-June 2024 (3 months), mid-September 2024 (6 months), and mid-March 2025 (12 months; same season as baseline). To minimize short-term rainfall effects, sampling was performed after ≥7 consecutive days without effective precipitation. All soil samples were collected from the planting area of a single cultivar (‘Yitong 2’). At each campaign, five soil cores (0–30 cm; 2.5-cm diameter auger) were collected within the plot using a five-point scheme and pooled to generate one composite sample. The composite was thoroughly homogenized (quartering method) and then split into three aliquots that were processed independently (DNA extraction/sequencing and physicochemical assays), providing technical replication for the analytical pipeline (*n* = 3 per time point; total *n* = 12 aliquots).

### 2.3. Experimental Materials

The plant material used in this study was *Idesia polycarpa* ‘Yitong 2’, a cultivar introduced from Tai’an, Shandong Province, and subsequently domesticated and selectively bred by the *I. polycarpa* research team of the College of Forestry, Henan Agricultural University. This cultivar exhibits a high fruit oil content of 41.45%, indicating substantial value for edible/industrial oil production. It also shows excellent fruiting performance, with a concentrated fruiting period from October to November; its berries are plump, with 20–110 fruits per infructescence. For trees aged 4–18 years, the fresh-fruit yield can reach approximately 600–2550 kg per mu, demonstrating stable productivity. The seeds are small yet well filled, which is favorable for propagation and subsequent processing and utilization. In addition, ‘Yitong 2’ has an open, well-structured crown and a regular growth form. The control cultivar in this experiment was ‘Yuji’ *I. polycarpa*, which was bred locally by the same research team and has been officially approved as an improved variety by Henan Province. It is a widely promoted high-quality cultivar in the region and is characterized by strong overall stress resistance and favorable economic traits, including pronounced tolerance to cold, drought, poor soils, and shading. This control cultivar was included to verify that ‘Yitong 2’ exhibited normal growth under the local conditions, thereby supporting the reliability of the subsequently collected experimental data.

### 2.4. Methods for Measuring Plant Phenotypes and Soil Physicochemical Properties

Measurements of *Idesia polycarpa* seedlings: A total of 960 *I. polycarpa* seedlings were planted in the experimental site, including 730 seedlings of ‘Yitong 2’ and 230 seedlings of the control cultivar ‘Yuji’. For growth measurements, 120 seedlings were randomly selected from each cultivar. Measurements were conducted in March 2024, June 2024, September 2024, and March 2025. All values used for subsequent analyses were expressed as means. The growth traits were measured as follows: plant height was measured with a measuring tape as the vertical distance from the soil surface to the terminal bud of the main stem, with a precision of 1 mm. Basal diameter was measured using a digital vernier caliper at the position of the main stem near the soil surface, with a precision of 0.1 mm. The number of lateral branches was determined by manual counting, including only living branches that emerged from the main stem, were normally developed, and were ≥5 cm in length.

The soil physicochemical properties were determined as follows [30,31,35]. Soil pH was measured using a pH meter after shaking a soil–water suspension (1:2.5, *m*:*v*) for 30 min. Soil water content (SWC) was determined by the oven-drying gravimetric method. Soil bulk density (SBD) was measured using the core method. Soil organic matter (SOM) was determined by the potassium dichromate oxidation method with external heating. Alkali-hydrolyzable nitrogen (AN) was measured using the alkaline hydrolysis–diffusion method. Available phosphorus (AP) was determined by the NaHCO_3_ extraction–molybdenum–antimony colorimetric method. Available potassium (AK) was determined by NH_4_OAc extraction followed by flame photometry. SWC was measured on fresh soil, whereas all other indices were measured using air-dried soil passed through a 2-mm sieve. To ensure data reliability, each index was measured in triplicate (*n* = 3), and the mean of the three measurements was used for statistical analyses. Soil pH: Approximately 10 g of air-dried, sieved soil was weighed and mixed with deionized water at a soil:water ratio of 1:2.5 (*m*:*v*) (10 g soil + 25 mL water). The suspension was shaken for 30 min and then allowed to stand until clarified. The pH of the supernatant was measured with a pH meter, with three parallel determinations per sample. Soil water content (SWC): Approximately 100 g of fresh soil was weighed and dried at 105 °C to constant weight. After cooling, the dry weight was recorded, and SWC was calculated based on mass loss. Each sample was measured in triplicate. Soil bulk density (SBD): SBD was determined using the core method. A standard 100 cm^3^ core ring was used to collect intact soil cores in the field. Samples were returned to the laboratory and dried at 105 °C to constant weight. Bulk density was calculated from the oven-dry mass and core volume, with three core replicates per plot. Soil organic matter (SOM): For each determination, 0.30 g of air-dried, sieved soil was weighed (preliminary tests indicated that 0.30 g provided reliable SOM quantification) and analyzed using the potassium dichromate oxidation method with external heating. Each sample was measured in triplicate. Alkali-hydrolyzable nitrogen (AN): Approximately 5.0 g of air-dried, sieved soil was weighed and analyzed using the alkaline hydrolysis–diffusion method, with three parallel determinations per sample. Available phosphorus (AP): Approximately 2.5 g of air-dried, sieved soil was extracted with NaHCO_3_, and phosphorus in the extract was determined using the molybdenum–antimony colorimetric method. Each sample was measured in triplicate. Available potassium (AK): Approximately 5.0 g of air-dried, sieved soil was extracted with NH_4_OAc, and potassium in the extract was measured by flame photometry. Each sample was measured in triplicate.

### 2.5. 16S rRNA Gene Sequencing

After removing the samples from the −80 °C refrigerator, a precise amount of 0.1–0.5 g of each sample was weighed immediately and transferred into a centrifuge tube containing extraction lysis buffer, followed by grinding treatment. For the pretreated samples, nucleic acids were extracted using the MagBeads FastDNA Kit for Soil (MP Biomedicals, CA, USA). The extracted DNA was subjected to 0.8% agarose gel electrophoresis to determine the molecular weight, and then quantified using a Nanodrop spectrophotometer. PCR amplification of the microbial community DNA in soil samples was performed using the universal primers 338F (5′-ACTCCTACGGGAGGCAGCA-3′) and 806R (5′-GGACTACHVGGGTWTCTAAT-3′) targeting the V3–V4 hypervariable regions of the bacterial 16S rRNA gene [36]. The reaction system was pre-denatured at 98 °C for 5 min, followed by 28 cycles of amplification (98 °C for 30 s, 55 °C for 45 s, 72 °C for 45 s), and finally extended at 72 °C for 5 min before being preserved at 12 °C. After verification by 2% agarose gel electrophoresis, the amplification products were detected again using 2% agarose gel electrophoresis. The target fragments were excised from the gel and recovered using the Axygen Gel Extraction Kit (New York, NY, USA). Subsequently, libraries were constructed with the TruSeq Nano DNA LT Library Prep Kit from Illumina (San Diego, CA, USA), and paired-end sequencing with a read length of 2 × 250 bp was performed using the NovaSeq 6000 SP Reagent Kit (500 cycles) (San Diego, CA, USA). The sequencing work was commissioned to Shanghai Personalbio Technology Co., Ltd. (Shanghai, China).

### 2.6. Data Processing and Analysis

The raw sequencing data were imported into a format compatible with QIIME2 (version 2022.11). The demux plugin was used for decoding, and the cut adapt plugin (version 2.3) was employed to remove primer sequences. Subsequent quality filtering, denoising, merging, and chimera removal were performed using the DADA2 plugin, with the expected error rates set to 2% for both forward and reverse reads and truncation lengths set to 223 bp and 230 bp, respectively. Through this pipeline, feature sequences (ASVs) and a corresponding abundance table were generated by clustering sequences at 100% similarity. For taxonomic assignment, the 16S rRNA gene sequences were aligned against the SILVA 138 database (at 99% similarity threshold). Visualization of sample composition distribution at the phylum and genus levels was conducted using QIIME2, displaying microbial community structure characteristics by relative abundance. Alpha diversity analysis was performed using QIIME2, R (version 3.6.1), and the ggplot2 package. For rarefaction curve analysis, samples were normalized by rarefying to 95% of the sample with the lowest sequencing depth prior to diversity analysis. ANOVA of soil alpha diversity was conducted using IBM SPSS 26.0. For beta diversity analysis, Principal Coordinate Analysis (PCoA) based on the Bray–Curtis distance algorithm was performed using QIIME2 software and the ape package in R (version 3.6.1). The functional potential of the soil microbial community was predicted using PICRUSt2 software and R (version 3.6.1) based on the ASVs obtained from 16S rRNA gene sequencing and the annotation results from the SILVA 138 database. Correlation heatmap analysis and Redundancy Analysis (RDA) were employed to explore the associations between the microbial community and soil physicochemical factors. Basic data were organized using Microsoft Excel 2019. Statistical analysis of soil physicochemical properties, alpha diversity indices, and metabolic pathways, including significance testing and correlation heatmap analysis, was performed using Origin 2024 and IBM SPSS 26.0, with related graphs generated accordingly.

## 3. Results

### 3.1. Analysis of Growth Status of Idesia polycarpa Seedlings at the Early Planting Stage

**Figure 2:**
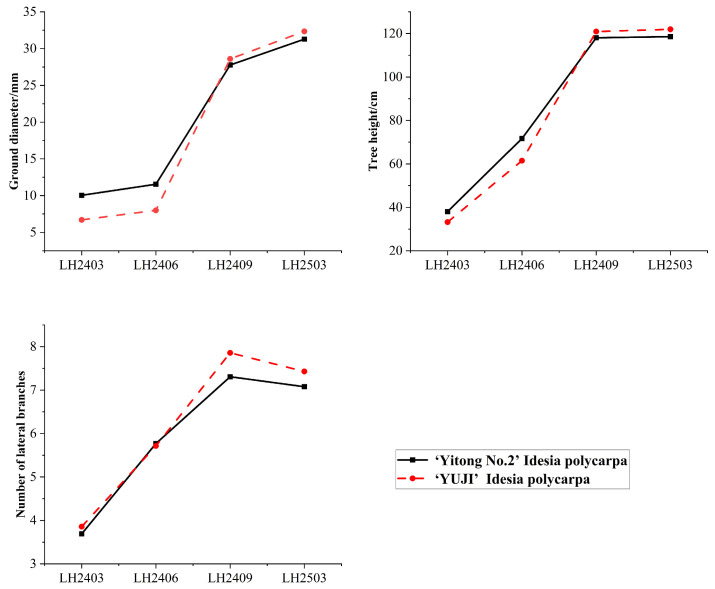
Growth variation trend of *Idesia polycarpa*. Note: In the figure, LH2403 represents March 2024, LH2406 represents June 2024, LH2409 represents September 2024, and LH2503 represents March 2025. The number of seedlings used for analysis is 120 plants, and the mean value is used for plotting and analysis.

After planting, both *Idesia polycarpa* cultivar ‘Yitong No.2’ and the approved elite cultivar ‘Yuji’ (used as the control) exhibited good adaptability (Figure 2). In terms of tree height, both cultivars kept growing rapidly. At the initial stage of planting (March 2024), the tree height of ‘Yitong No.2’ was 38.05 cm, while that of ‘Yuji’ was 33.21 cm. By the end of the growing season (September 2024) and the same period of the following year (March 2025), the tree heights of both cultivars reached a similar level of approximately 120 cm. This indicated that there was no significant difference in tree height growth potential between the two cultivars, and both showed favorable growth performance under the given site conditions. The growth dynamics of ground diameter showed that both cultivars exhibited a growth pattern of slow thickening in the early stage (from March 2024 to June 2024) followed by rapid growth in the later stage (after September 2024). Although the ground diameter of ‘Yitong No.2’ (10.05 mm) was slightly larger than that of ‘Yuji’ (6.71 mm) at the initial stage of planting, both cultivars achieved significant radial growth during the subsequent observation period. After one year of growth, their ground diameters increased to 31.29 mm and 32.34 mm, respectively, by March 2025, with similar growth multiples. There was no significant difference in the number of lateral branches between the two cultivars; the number of lateral branches increased steadily from approximately 3–4 at the initial planting stage to more than 7 at the end of the observation period, reflecting the normal development and expansion of the canopy structure. The above analysis shows that *Idesia polycarpa* saplings grew normally during the early planting period.

### 3.2. Analysis of Changes in Soil Physicochemical Properties After Idesia polycarpa Planting

Analysis of soil physicochemical properties in *Idesia polycarpa* plantations at different sampling times, shown in Table 1, found that some soil physicochemical properties exhibited significant changes over time after planting (*p* < 0.05). The content of soil available potassium (AK) showed a continuous and highly significant accumulation trend, increasing from 96.35 mg/kg before planting to 150.55 mg/kg one year after planting (*p* < 0.05), an increase of 56.2%. Soil water content (SWC) presented obvious seasonal fluctuations: it was relatively low in the peak plant growth periods and high-temperature seasons (June and September 2024), reaching 11.22% and 8.50%, respectively. Soil organic matter (SOM) content exhibited a dynamic change of initial decrease followed by recovery, reaching the highest value of 19.04 g/kg in March 2024; it then decreased significantly to 15.20 g/kg in June 2024 (*p* < 0.05). Subsequently, SOM content recovered slightly in September 2024 and March 2025, with values of 16.97 g/kg and 18.19 g/kg, respectively, showing no significant difference from the pre-planting levels (*p* > 0.05). Soil pH remained relatively stable, with no significant differences observed across all sampling periods (*p* > 0.05). The responses of soil available nutrients to planting varied to some extent: the contents of alkaline-hydrolyzable nitrogen (AN) and available phosphorus (AP), as well as soil bulk density (SBD), fluctuated across different periods but without significant differences (*p* > 0.05).

### 3.3. Analysis of Changes in ASVs of Soil Microbial Communities After Idesia polycarpa Planting

**Figure 3:**
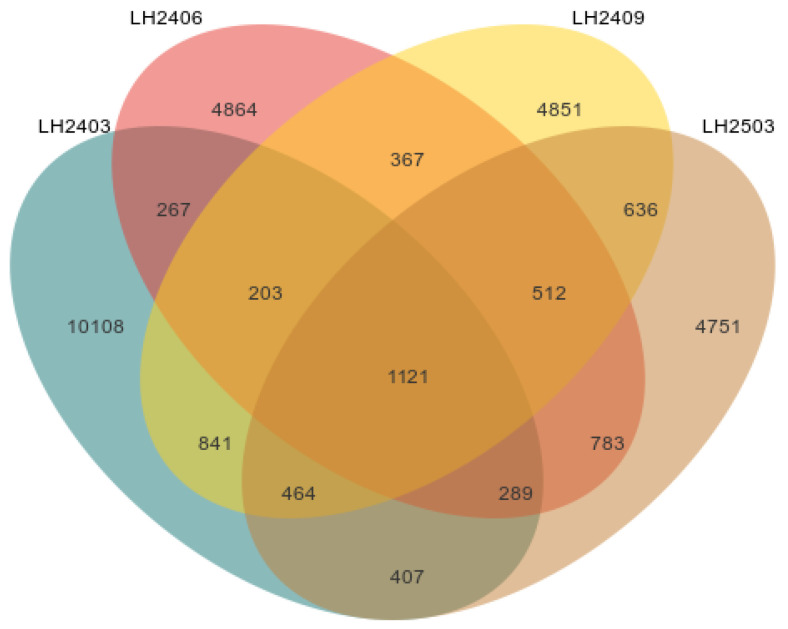
Distribution of soil ASV numbers in different sampling periods. Note: In the figure, LH2403 represents March 2024, LH2406 represents June 2024, LH2409 represents September 2024, and LH2503 represents March 2025. Each sampling node has 3 repetitions, and the mean value is used for analysis.

High-throughput sequencing of the obtained samples was performed using the QIIME2 platform. As shown in the Venn diagram (Figure 3), a total of 25,701 ASVs (amplicon sequence variants) were identified across the four time points. Among them, 407 ASVs were shared across all four time points, with varying degrees of overlap between groups, including pairwise shared ASVs (e.g., 267 ASVs shared between March 2024 and June 2024) and ASVs shared by three time points (e.g., 1121 ASVs shared between June 2024, September 2024, and March 2025). Overall, the four periods shared a certain number of ASVs, indicating a fundamental commonality in community composition. However, many unique ASVs were also observed, suggesting significant differences in community composition at different stages, which is consistent with the changes in community structure over time.

### 3.4. Analysis of Changes in Soil Microbial Community Diversity After Idesia polycarpa Planting

To investigate the changes in soil bacterial diversity after *Idesia polycarpa* planting, α diversity indices across different sampling periods were analyzed, with the results presented in Table 2. The α diversity of soil bacterial communities was significantly altered after *Idesia polycarpa* planting. Before planting (March 2024), the soil bacterial species richness (Chao1 index) reached the highest value (5709.14 ± 648.74). After planting, the Chao1 index decreased significantly (*p* < 0.05), dropping to 3656.08 ± 64.64 in June 2024, and exhibited no significant changes thereafter (*p* > 0.05). Meanwhile, the community evenness (Pielou e index) exhibited an opposite trend of change. The evenness was the lowest before planting (0.8875 ± 0.0060), and the values recorded in the subsequent three observation periods were significantly higher than that at the initial stage (*p* < 0.05). The Shannon index, which reflects both species richness and community evenness, showed no significant differences between sampling periods (*p* > 0.05).

**Figure 4:**
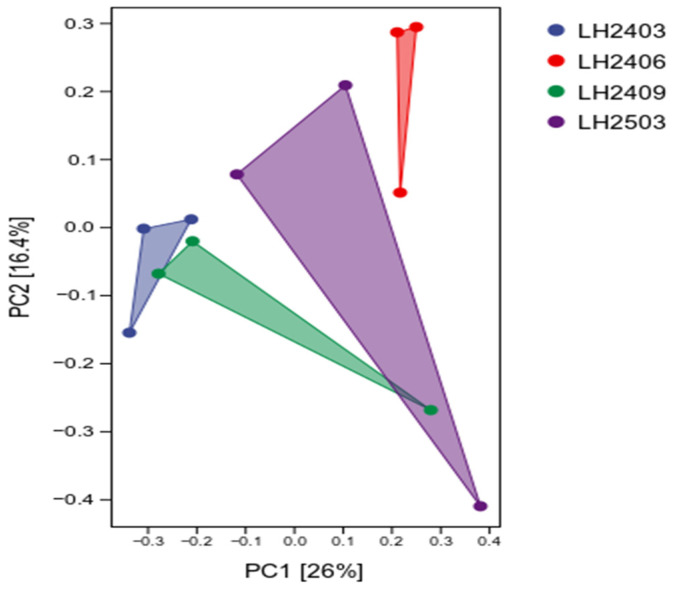
PCoA of soil bacterial communities based on Bray–Curtis distances. Note: Each point represents one technical replicate aliquot (*n* = 3 per time point). Polygons indicate the convex hull for each sampling period. PERMANOVA and beta-dispersion results are reported in the main text. In the figure, LH2403 represents March 2024, LH2406 represents June 2024, LH2409 represents September 2024, and LH2503 represents March 2025. Each sampling node has 3 repetitions, and the mean value is used for analysis.

To investigate the effects of *Idesia polycarpa* planting on soil microbial β diversity, Principal Coordinate Analysis (PCoA) was performed, and the results revealed that *Idesia polycarpa* planting exerted a certain impact on the soil bacterial community structure (Figure 4). Axis 1 and Axis 2 explained 26.0% and 18.7% of the total variation, respectively. Observations showed that samples exhibited a high degree of aggregation and small differences in soil bacterial community structure at the initial planting stage. With the passage of time, during the September 2024 and March 2025 periods, the degree of sample aggregation decreased, and the dispersion degree showed a gradually increasing trend. To verify whether the grouping differences observed in PCoA were statistically significant, we conducted a PERMANOVA test based on Bray–Curtis distances (999 permutations). The results indicated that the sampling time had a significant effect on bacterial community structure (PERMANOVA: Pseudo-F = 1.5084, R^2^ = 0.3613, *p* = 0.024). Additionally, a beta dispersion analysis (BETA DISP) was performed to test for differences in within-group dispersion (F = 0.3581, *p* = 0.778), which helped rule out potential effects of within-group dispersion differences on the PERMANOVA results.

### 3.5. Composition of Soil Microbial Communities After Idesia polycarpa Planting

**Figure 5:**
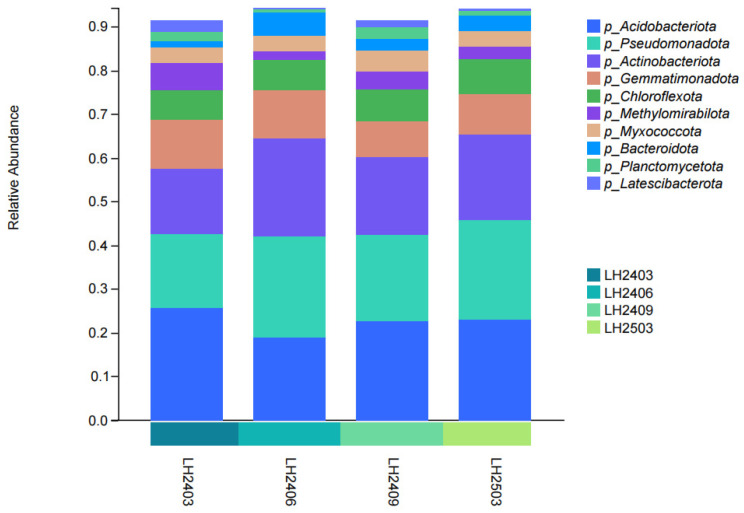
Relative abundance of dominant bacterial phyla across sampling periods. Note: In the figure, LH2403 represents March 2024, LH2406 represents June 2024, LH2409 represents September 2024, and LH2503 represents March 2025. Each sampling node has 3 repetitions, and the mean value is used for analysis.

After taxonomic classification of soil bacteria, results showed that at the phylum level, among the top 10 taxa in terms of relative abundance (Figure 5), the soil bacterial communities across all sampling periods were dominated by three major phyla: Acidobacteriota, Pseudomonadota (formerly Proteobacteria), and Actinobacteriota. The combined relative abundance of these three phyla accounted for more than 55% of the total bacterial community. However, the relative abundances of these three phyla varied with planting time. Specifically, the relative abundance of Pseudomonadota (formerly Proteobacteria) exhibited an increasing trend after planting, reaching the highest level in June 2024. On the contrary, the relative abundance of Acidobacteriota was the highest before planting (March 2024), then decreased significantly and stabilized thereafter. In addition, Actinobacteriota and other dominant phyla including Chloroflexota and Gemmatimonadota also underwent dynamic changes, though to varying degrees.

**Figure 6:**
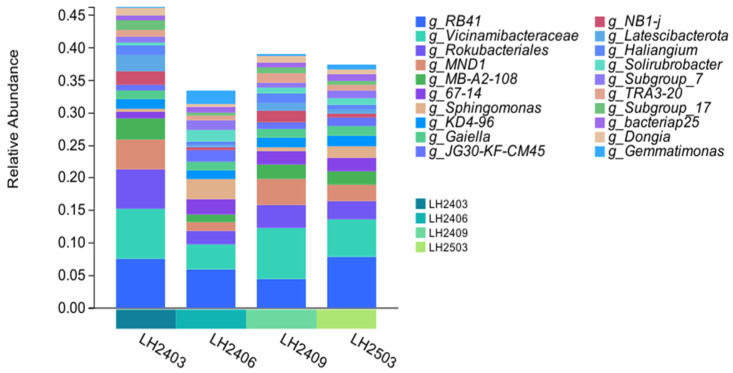
Relative abundance of dominant bacterial genera across sampling periods. Note: In the figure, LH2403 represents March 2024, LH2406 represents June 2024, LH2409 represents September 2024, and LH2503 represents March 2025. Each sampling node has 3 repetitions, and the mean value is used for analysis.

Analysis of changes in soil bacterial communities at the genus level after *Idesia polycarpa* planting revealed corresponding shifts in community structure (Figure 6). Among the top 20 bacterial genera in terms of relative abundance, their total relative abundance before planting was higher than that after planting. Specifically, their total relative abundance dropped to the lowest level at three months after planting (June 2024); although it showed a recovery afterward, it still differed to a certain extent from the pre-planting level. Compared with the pre-planting period, several genera including *Vicinamibacteraceae*, *Rokubacteriales*, *MND1*, *MB-A2-108*, *NB1-j*, and *Latescibacterota* exhibited a significant decreasing trend in relative abundance with a substantial reduction magnitude. In contrast, the relative abundances of genera such as *67-14*, *Solirubrobacter*, *Subgroup_7*, and *Gemmatimonas* increased significantly compared with the pre-planting level. The genus *RB41* showed a trend of initial decrease followed by subsequent increase.

### 3.6. Analysis of Differences in Soil Microbial Community Structure After Idesia polycarpa Planting

**Figure 7:**
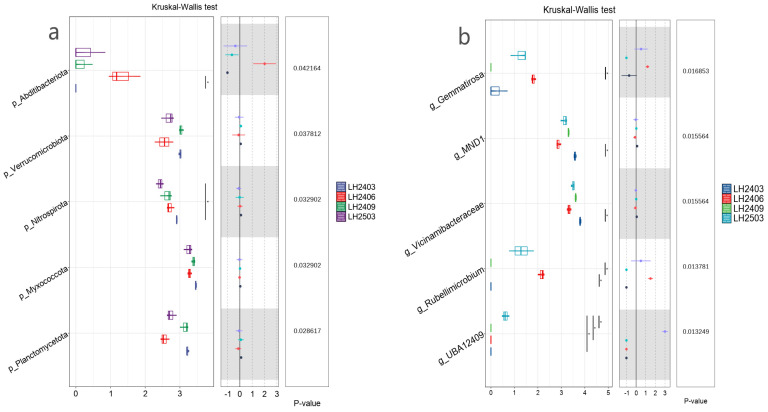
Differential taxa among sampling periods at the phylum (**a**) and genus (**b**) levels. Note: Boxplots show distributions across technical replicates (*n* = 3 per time point). Group differences were assessed using the Kruskal–Wallis test with FDR correction (*p* values shown). In the figure, LH2403 represents March 2024, LH2406 represents June 2024, LH2409 represents September 2024, and LH2503 represents March 2025 (* *p* < 0.05).

The Kruskal–Wallis test was used to compare the abundance differences of each taxonomic unit among sample groups one by one. After *Idesia polycarpa* planting, the soil bacterial communities exhibited significant stage-specific differences at both the phylum and genus levels (Figure 7), and the top 5 species with significant differences were selected for visualization.

At the phylum level (Figure 7a), the differences in community structure were mainly reflected in the comparison between the pre-planting period (March 2024) and the subsequent periods (June 2024, September 2024, March 2025). Multiple comparative analysis revealed that five phyla, namely Planctomycetota (*p *= 0.0286), Myxococcota (*p* = 0.0329), Nitrospirota (*p* = 0.0329), Verrucomicrobiota (*p *= 0.0378), and Abditibacteriota (*p* = 0.0422), exhibited the most significant differences across different sampling periods (*p *< 0.05). The relative abundances of Planctomycetota and Verrucomicrobiota showed an obvious trend of initial decrease followed by recovery. The relative abundance of Nitrospirota exhibited a significant decrease during the three post-planting sampling periods (June 2024, September 2024, March 2025) (*p* < 0.05). In addition, the relative abundance of Abditibacteriota showed a significant difference between the pre-planting period and the stage at three months after planting (June 2024).

At the genus level (Figure 7b), significant differences were also observed, with the genera *UBA12409*, *Rubellimicrobium*, *Vicinamibacteraceae*, *MND1*, and *Gemmatirosa* ranking among the top 5 in terms of significant differences. The relative abundance of UBA12409 reached the highest level in the 4th post-planting sampling period (March 2025), showing a significant advantage over the previous three periods (*p* < 0.05). Both *Rubellimicrobium* and *Gemmatirosa* achieved their peak relative abundances in the 2nd sampling period (June 2024); however, the relative abundance of *Rubellimicrobium* was significantly higher than those in the March 2024 and March 2025 periods, while that of *Gemmatirosa* was significantly higher than that in the September 2024 period.

### 3.7. Correlation Analysis Between Soil Physicochemical Properties and Soil Bacterial Community Structure

**Figure 8:**
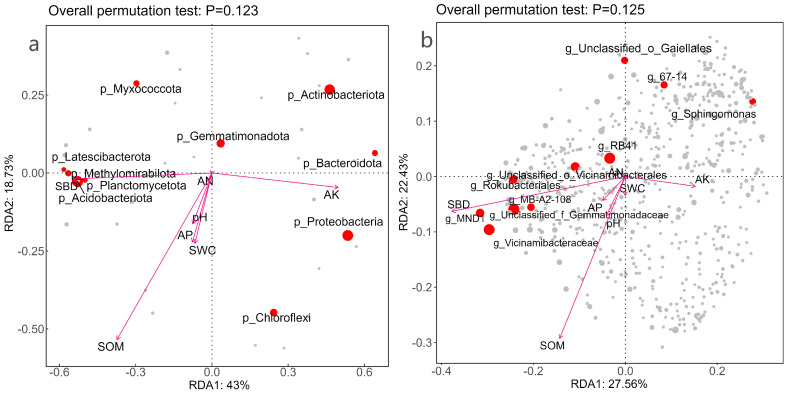
Correlation analysis between soil physicochemical properties and bacterial community structure. Note: (**a**) represents the phylum level, (**b**) represents the genus level. Abbreviations: AK, quick-acting potassium; SWC: soil water content; pH: power of hydrogen; SBD: soil bulk density; AP, effective phosphorus; AN: alkali dissolved nitrogen; SOM, total organic matter. In the figure, LH2403 represents March 2024, LH2406 represents June 2024, LH2409 represents September 2024, and LH2503 represents March 2025. Each sampling node is set with 3 replicates, and the mean value is used for analysis.

Redundancy analysis (RDA) was used to explore associations between community composition and soil variables. At the phylum level (Figure 8a), RDA1 and RDA2 explained 43.0% and 18.7% of the constrained variation, respectively; however, the overall model was not significant in permutation tests (*p* = 0.123). Among individual variables, soil organic matter showed a significant marginal association with the ordination (envfit: r^2^ = 0.522, *p* = 0.036), whereas available potassium and soil bulk density showed moderate but non-significant associations (*p* > 0.05). At the genus level (Figure 8b), the overall model was also not significant (*p* = 0.125); soil bulk density and soil organic matter showed the strongest associations with compositional variation. Together, these analyses suggest that within this fixed site, temporal changes in SOM and soil structure may be linked to community turnover.

At the genus level, as shown in Figure 8b, Axis 1 and Axis 2 together explained approximately 49.99% of the variation in community composition. In contrast to the results at the phylum level, both soil bulk density (SBD) and soil organic matter (SOM) exerted significant effects on the bacterial community composition at the genus level (SBD: r^2^ = 0.618, *p* = 0.009; SOM: r^2^ = 0.525, *p* = 0.027). SBD was the most powerful explanatory factor, and its vector pointed primarily along the RDA1 axis, indicating that soil physical structure (bulk density) was the primary environmental variable affecting the distribution differences of bacterial genera (e.g., RB41, Vicinamibacteraceae, and unclassified Gaiellales). SOM also exerted a significant effect, and its direction of action was more closely associated with the RDA2 axis. Other nutrient factors also had a certain degree of influence, but their effects were relatively weak and non-significant.

### 3.8. Analysis of Dynamic Differences in Soil Microbial Functions After Idesia polycarpa Planting

**Figure 9:**
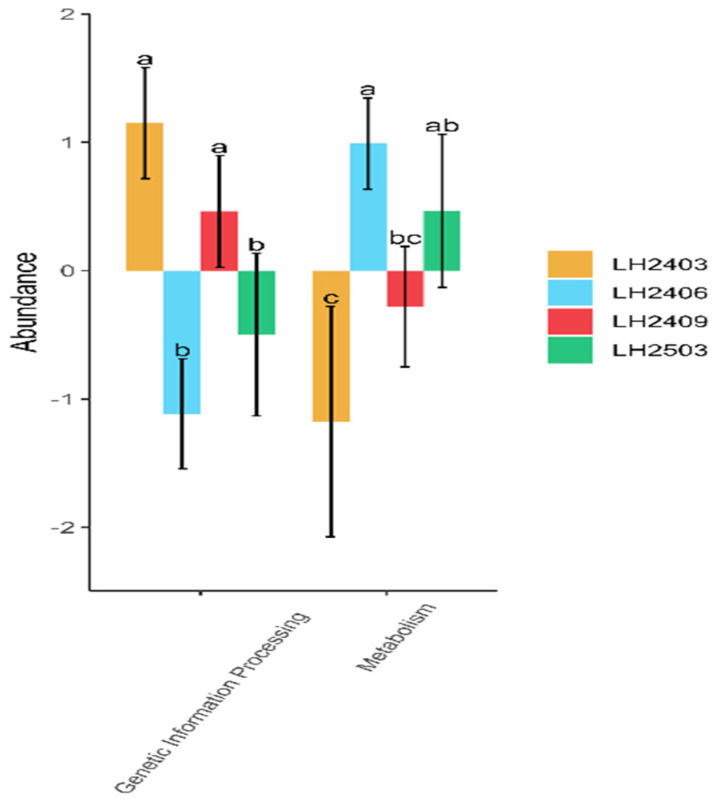
Differences in predicted KEGG level-1 functional categories (PICRUSt2) across sampling periods. Note: In the figure, LH2403 represents March 2024, LH2406 represents June 2024, LH2409 represents September 2024, and LH2503 represents March 2025. Each sampling node has 3 repetitions, and the mean value is used for analysis. The letters in the figure indicate significant differences among groups (*p* < 0.05), with the maximum value labeled as “a”.

Functional potential was inferred using PICRUSt2 and summarized using the KEGG hierarchy. Across sampling periods, the overall predicted functional architecture was broadly stable. At KEGG level 1, six top-level categories were detected (Cellular Processes, Environmental Information Processing, Genetic Information Processing, Human Diseases, Metabolism, and Organismal Systems). Because KEGG is a pathway annotation framework that includes host-oriented categories, the presence of labels such as ‘Human Diseases’ reflects database organization rather than implying human disease processes in soil; therefore, we focus interpretation on ecology-relevant categories (primarily Metabolism and Genetic Information Processing). Significant temporal differences were detected for Genetic Information Processing and Metabolism (Kruskal–Wallis, *p* < 0.05; Table 3; Figure 9). Genetic Information Processing showed higher relative abundance in March 2024 and September 2024, whereas Metabolism peaked in June 2024. These patterns suggest that shifts in community composition were accompanied by modest adjustments in predicted functional potential during the first year of plantation establishment.

**Figure 10:**
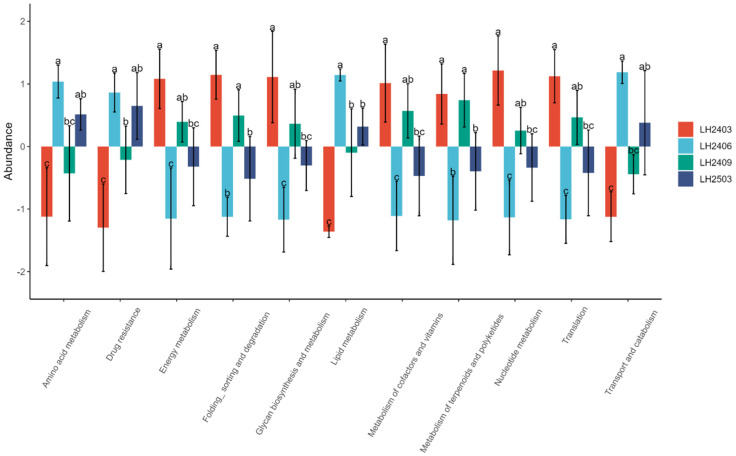
Differences in selected predicted KEGG level 2 pathways (PICRUSt2) across sampling periods. Note: In the figure, LH2403 represents March 2024, LH2406 represents June 2024, LH2409 represents September 2024, and LH2503 represents March 2025. Each sampling node has 3 repetitions, and the mean value is used for analysis. The letters in the figure indicate significant differences among groups (*p* < 0.05), with the maximum value labeled as “a”.

In the analysis of second-level metabolic pathways, a total of 29 metabolic pathways were detected. Multiple comparative analysis of these metabolic pathways revealed (Figure 10) that 11 pathways exhibited significant differences (*p* < 0.05). The pathways of Amino acid metabolism, Drug resistance, Lipid metabolism, and Transport and catabolism showed significantly higher relative abundances at three months after planting (June 2024) and one year after planting (March 2025) compared with those in the pre-planting period (March 2024). For the other 7 metabolic pathways, their relative abundances in the pre-planting period (March 2024) and at six months after planting (September 2024) were significantly higher than those in the other two periods. The 11 second-level metabolic pathways were classified into 4 first-level metabolic pathways. Specifically, Amino acid metabolism, Energy metabolism, Glycan biosynthesis and metabolism, Lipid metabolism, Metabolism of cofactors and vitamins, Metabolism of terpenoids and polyketides, and Nucleotide metabolism belong to the first-level metabolic pathway of Metabolism; Folding, sorting and degradation and Translation belong to the first-level metabolic pathway of Genetic Information Processing; Transport and catabolism belongs to the first-level metabolic pathway of Cellular Processes; and Drug resistance belongs to the first-level metabolic pathway of Human Diseases. The differences in the second-level metabolic pathways were mainly concentrated in the Metabolism pathway.

## 4. Discussion

### 4.1. Growth Trends of Idesia polycarpa and Effects of Changes in Soil Physicochemical Properties After Planting

Different vegetation cover types have a significant impact on the formation of soil physicochemical properties [37]. The results of this study showed that significant changes occurred in soil physicochemical properties within one year after planting *Idesia polycarpa* in the Luohe River Basin. Among these changes, the content of soil available potassium (AK) showed a continuous and extremely significant accumulation trend. This might be attributed to the fact that *Idesia polycarpa*, as a fast-growing tree species, its root exudates, root activities, and litter input catalyzed the decomposition of organic matter [38,39], thereby promoting the accumulation of available potassium in the soil. The content of soil organic matter (SOM) exhibited a dynamic change characteristic of initial decrease followed by recovery. This was because in the initial stage after planting, soil microorganisms decomposed and consumed a large amount of original organic matter; meanwhile, the plants were still in the early growth stage, with limited input of litter and exogenous carbon sources, leading to an unbalanced state where organic matter consumption exceeded input. As plant growth stabilized in the later stage, litter return increased, supplemented by carbon source input from root exudates, and thus the soil organic matter content gradually recovered [40]. In addition, other indicators exhibited no significant changes within one year. The indicators that showed significant differences during the year also returned to normal levels one full year after planting. No significant differences were observed when comparing the pre-planting period (March 2024) with the one-year post-planting period (March 2025). The stability of soil bulk density (SBD) also indicated that under the current tending and management practices, the planting activity had not caused significant compaction or loosening of the soil physical structure.

### 4.2. Effects of Idesia polycarpa Planting on Soil Bacterial Community Structure and Diversity

Soil bacterial community diversity is significantly affected by multiple factors following plant cultivation, including plant growth, soil type, and pre-planting land use patterns, and the variation trends differ across different plant growth stages [41,42,43]. Alpha diversity analysis showed that after planting *Idesia polycarpa*, the species richness (Chao1 index) of soil bacterial communities decreased significantly (*p* < 0.05), while the community evenness (Pielou e index) increased significantly (*p* < 0.05); in contrast, the Shannon diversity index remained stable. This indicated that planting *Idesia polycarpa* did not simply reduce the overall diversity, but rather eliminated species that were not adapted to the new rhizosphere environment, and simultaneously enabled the remaining species to achieve more balanced competition and utilization of resources, thus forming a new microbial community characterized by lower richness, higher evenness, and stable overall diversity [44,45].

Principal Coordinate Analysis (PCoA) of Beta diversity further confirmed that the soil bacterial community structure underwent distinct changes after planting *Idesia polycarpa* compared with that in the pre-planting period (March 2024). Moreover, with the passage of time, the dispersion degree of community structure between the pre-planting period (March 2024) and the one-year post-planting period (March 2025) increased continuously, indicating that the bacterial community was undergoing succession toward a new state [46]. In terms of taxonomic composition, the absolute dominant taxa at the phylum level remained unchanged, still dominated by Acidobacteriota, Pseudomonadota, and Actinobacteriota; however, their relative abundances exhibited dynamic changes with planting time. This might be attributed to the fact that soil microbial composition is relatively similar in small-scale environments [47], while the planting of *Idesia polycarpa* served as the key driving factor triggering changes in the relative abundances of the dominant phyla. Pseudomonadota has the functions of promoting plant growth, nitrogen fixation, and enhancing soil fertility, and its increased abundance is conducive to accelerating soil nutrient cycling [48]. As a newly classified bacterial phylum, Acidobacteriota has been used as an indicator of changes in soil nutrient status [49]; it can drive material cycling and ecological environment construction in soil. Both phyla play a role in improving the soil rhizosphere environment [50]. In addition, taxa such as Pseudomonadota and Actinobacteriota also possess heterotrophic physiological functions, and play important roles in carbon metabolism and assimilation, elemental biogeochemical cycling, organic matter turnover, and degradation of complex organic compounds [51]. Changes at the genus level were more specific: the abundances of genera such as *Vicinamibacteraceae* and *MND1* decreased, while those of genera including *RB41*, *Subgroup_7*, and *Gemmatimonas* increased. Differential species identified by the Kruskal–Wallis test, including the phyla Planctomycetota and Nitrospirota as well as the genera *UBA12409* and *Rubellimicrobium*, further defined the key sensitive taxa responding to the planting activity. The turnover of these taxa not only revealed the shift in microbial community functions, but also indicated that the planting of *Idesia polycarpa* had driven the soil bacterial community to undergo directional and regular succession. This might be associated with the root exudates of *Idesia polycarpa*: by regulating the metabolic characteristics of root exudates to modulate the structural characteristics of soil bacterial communities, it ultimately enabled the entire microbial community to adapt to the new post-planting environment [52,53,54].

### 4.3. Effects of Soil Physicochemical Properties and Plant Growth on Soil Microbial Community Structure

Redundancy Analysis (RDA) revealed the close association between soil physicochemical factors and microbial community structure, among which, soil organic matter (SOM) and soil bulk density (SBD) were the primary factors affecting changes in microbial community structure, exerting a profound impact on the distribution pattern and taxon abundance of soil microorganisms [55]. From the perspective of the relationship between soil physicochemical properties and soil bacterial phyla and genera over one year (March 2024–March 2025), at the phylum level, soil organic matter (SOM) was the most significant environmental factor driving variations in community structure. This is consistent with the core role of organic matter as an energy and carbon source for microorganisms, and the dynamic changes in its content directly affect the prosperity and decline of microbial taxa dependent on different carbon sources [56,57]. At the genus level, soil bulk density (SBD) and soil organic matter (SOM) jointly exerted a significant regulatory effect on the community development of bacterial genera. This result also indicated that after planting *Idesia polycarpa*, the growth of plant roots can indirectly affect soil microbial community structure by altering soil physicochemical properties [58].

The *Idesia polycarpa* cultivar (‘Yitong No. 2’) exhibited a sound growth performance throughout the observation period, with both tree height and ground diameter maintaining a steady increase, and its growth level was comparable to that of the improved control variety. This favorable growth status ensures that the observed changes in soil bacterial communities in this study can be reasonably attributed to vegetation introduction and the accompanying ecological processes, rather than stress effects caused by inhibited plant growth. The normal growth of plants sustains the occurrence of biological processes such as continuous root exudate input, fine root turnover, and potential mycorrhizal symbiosis. These biological processes act synergistically with the aforementioned changes in soil physicochemical factors (e.g., SOM dynamics and AK accumulation), jointly shaping a new rhizosphere microbial environment and further driving the directional succession of the community structure.

### 4.4. Effects of Idesia polycarpa Planting on Soil Microbial Metabolic Functional Pathways

Based on the analysis of metabolic pathways over one year as well as between the pre-planting stage (March 2024) and post-planting stage (March 2025), combined with PICRUSt2 functional prediction and KEGG database annotation, it was shown that the overall functional architecture of soil microbial communities remained stable after planting *Idesia polycarpa*, with only some metabolic pathways exhibiting significant differences. This indicated that *Idesia polycarpa* planting exerted a certain impact on soil bacterial metabolic pathways, but the impact was relatively minor. At the level of first-level metabolic pathways, differences mainly manifested between the pre-planting stage (March 2024) and post-planting stages (June 2024, September 2024, March 2025). In contrast, for second-level metabolic pathways, all pathways with significant differences showed notable variations between the pre-planting stage (March 2024) and one year after planting (March 2025). Specifically, among the first-level metabolic pathways, the relative abundances of the Genetic Information Processing and Metabolism pathways exhibited significant variations over one year (*p* < 0.05). Among these, the Metabolism pathway serves as the primary core function within the bacterial community [59], and its subordinate second-level metabolic pathways, including Amino acid metabolism, Energy metabolism, and Metabolism of cofactors and vitamins, are the main functions of the bacterial community [60] and also constitute the primary regions where differences were concentrated. Amino acid metabolism facilitates the absorption and utilization of amino acids by bacteria, providing a fundamental guarantee for their growth and reproduction [61]. In contrast, Energy metabolism and Metabolism of cofactors and vitamins are directly related to material transformation and the maintenance of physiological activity within the bacterial community [60], which also reflects the functional adaptation of microorganisms to changes in the rhizosphere nutrient environment. In addition, the differences in the second-level metabolic pathways within the Genetic Information Processing pathway reflect the adjustments made by soil microbial communities in functions such as protein synthesis and repair to adapt to the new environment. These changes in functional potential may be related to the alterations in soil environments following *Idesia polycarpa* planting. To adapt to the new environment, the soil ecosystem modifies the composition of soil bacteria, and the metabolic pathways respond accordingly, thereby making the entire soil ecosystem more conducive to the growth and development of *Idesia polycarpa*.

## 5. Conclusions

This study systematically analyzed the dynamic changes in soil microbial communities and physicochemical properties, as well as their feedback relationships, following the planting of *Idesia polycarpa* in the Luohe River Basin over a one-year field-based monitoring period. The results showed that *I. polycarpa* grew well during the early stages of planting, with significant accumulation of available potassium in the soil and a dynamic balance of organic matter that first declined and then recovered. The bacterial community richness (Chao1 index) significantly decreased from 5709.14 to 3656.08 (*p* < 0.05), while the evenness (Pielou e index) significantly increased from 0.8875 to 0.9216 (*p* < 0.05), and Shannon diversity remained stable. The community structure underwent directional succession, with an increase in the relative abundance of Pseudomonadota and a decrease in Acidobacteriota. Redundancy analysis revealed that soil organic matter and bulk density were the key factors driving microbial community structure changes. Functional prediction showed that the overall microbial metabolic framework remained stable, but some pathways (e.g., genetic information processing and metabolism) underwent significant adjustments. The findings provide theoretical support for soil ecological management and sustainable operation of *I. polycarpa* plantations. Due to site and research limitations, this study only focused on the changes in soil physicochemical properties and microbial communities within one year. Moreover, since this study is based on 16S rRNA amplicon sequencing and functional prediction through classification inference, it is difficult to directly reflect functional genes and enzymatic activity, and may overestimate pathway differences. In future research, we will combine this study’s findings with multi-year observations and multi-omics techniques to further elucidate long-term ecological effects and mechanisms.

## Figures and Tables

**Table 1:** Differences in soil physicochemical properties of *Idesia polycarpa* plantations across different sampling periods.

Index	Mar 2024	Jun 2024	Sep 2024	Mar 2025
pH	6.97 ± 0.11 ^a^	6.92 ± 0.22 ^a^	7.09 ± 0.11 ^a^	7.01 ± 0.15 ^a^
SOM g/kg^−1^	19.04 ± 1.88 ^a^	15.20 ± 1.27 ^b^	16.97 ± 1.89 ^ab^	18.19 ± 2.00 ^ab^
AN mg/kg^−1^	70.91 ± 23.10 ^a^	65.18 ± 5.69 ^a^	67.79 ± 1.85 ^a^	70.01 ± 1.52 ^a^
AP mg/kg^−1^	18.97 ± 3.67 ^a^	15.25 ± 3.21^a^	18.99 ± 5.35 ^a^	21.58 ± 2.87 ^a^
AK mg/kg ^1^	96.35 ± 15.31 ^b^	142.99 ± 5.42 ^a^	146.88 ± 7.57 ^a^	150.55 ± 4.41^a^
SWC /%	15.21 ± 3.98 ^ab^	11.22 ± 1.55 ^bc^	8.50 ± 1.34 ^c^	17.69 ± 0.76 ^a^
SBD g/cm^3^	1.27 ± 0.11 ^a^	1.08 ± 0.05 ^a^	1.36 ± 0.08 ^a^	1.25 ± 0.14 ^a^

Note: Data are presented as mean ± SD (*n* = 3 technical replicates per sampling period). Different lowercase letters indicate significant differences among sampling periods (*p* < 0.05). Abbreviations: AK, available potassium; AP, available phosphorus; AN, alkali-hydrolyzable nitrogen; SOM, soil organic matter; SBD, soil bulk density; SWC, soil water content.

**Table 2:** Alpha diversity analysis of soil bacteria in different sampling periods.

Sampling Period	Chao1 Exponents	Pielou e Exponents	Shannon Exponents
Mar 2024	5709.14 ± 648.74 ^a^	0.8875 ± 0.0060 ^b^	10.9430 ± 0.2040 ^a^
Jun 2024	3656.08 ± 64.644 ^b^	0.9216 ± 0.0027 ^a^	10.8749 ± 0.0294 ^a^
Sep 2024	3612.69 ± 459.69 ^b^	0.9153 ± 0.0116 ^a^	10.7877 ± 0.3016 ^a^
Mar 2025	3637.83 ± 998.40 ^b^	0.9135 ± 0.0310 ^a^	10.8454 ± 0.0568 ^a^

Note: Data are presented as mean ± SD (*n* = 3 technical replicates per sampling period). Different lowercase letters indicate significant differences among sampling periods (*p* < 0.05).

**Table 3:** Predicted KEGG level 1 functional categories of soil bacterial communities across sampling periods.

Metabolic Pathway	Mar 2024	Jun 2024	Sep 2024	Mar 2025
Cellular Processes	1796.44	1805.81	1777.88	1777.26
Environmental Information Processing	836.418	838.658	838.780	846.817
Genetic Information Processing	5641.85	5230.54	5495.46	5312.1
Human Diseases	85.6472	96.8471	97.2793	96.6244
Metabolism	35,803.3	35,605.8	35,660.1	35,481.3
Organismal Systems	173.165	114.79	124.828	121.172

Note: Values are mean ± SD across technical replicates (*n* = 3) for each sampling period.

## Data Availability

The original data presented in the study are openly available in the NCBI Sequence Read Archive (SRA) under Bio Project accessions PRJNA1264484 (March 2024 baseline) and PRJNA1420015 (June 2024, September 2024 and March 2025).
